# Metabolic Niches and Plasticity of Sand-Dune Plant Communities Along a Trans-European Gradient

**DOI:** 10.3390/metabo15040217

**Published:** 2025-03-24

**Authors:** Matthew P. Davey, Rachel M. George, Mark K. J. Ooi, Mike M. Burrell, Robert P. Freckleton

**Affiliations:** 1 Scottish Association for Marine Science (SAMS), Oban, Argyll PA37 1JQ, UK; 2 Ecology and Evolutionary Biology, School of Biosciences, University of Sheffield, Sheffield S10 2TN, UKr.freckleton@sheffield.ac.uk (R.P.F.); 3 School of Biological, Earth and Environmental Sciences, UNSW Sydney, Sydney, NSW 2052, Australia; mark.ooi@unsw.edu.au

**Keywords:** sand dunes, metabolomics, plasticity, climate change

## Abstract

**Background:** One of the greatest challenges to biologists is to understand the adaptive mechanisms of how plants will respond to climate at all levels from individual physiology to whole populations. For example, variation (plasticity) in the composition and concentration of metabolites will determine productivity, reproduction, and ultimately survival and distribution of plants, especially those subjected to rapid climate change. **Objectives:** Our aim was to study how interspecific and intraspecific metabolic variation in plant species within a single community can be elucidated. **Methods:** We used a metabolomics approach to study metabolic acclimation (by measuring the metabolome between plants under “common garden” controlled environment conditions) and metabolic plasticity (using field based reciprocal transplant studies) in a set of Atlantic sand dune annual communities along a latitudinal gradient from Portugal to England. **Results:** In the common garden study, metabolically phenotyping (using a fingerprinting direct injection mass spectrometry approach) five species of annual plants showed that species living together in a community have distinct metabolic phenotypes (high inter-specific metabolic variation). There was low intra-specific metabolic variation between populations growing under standard environmental conditions. The metabolic variation in one species *Veronica arvensis* was measured in the reciprocal transplant study. Metabolic phenotypes obtained from all samples were similar across all sites regardless of where the plants originated from. **Conclusions:** This implies that the metabolome is highly plastic and the measurable metabolome in this study was influenced more by local environmental factors than inherent genetic factors. This work highlights that species are fulfilling different niches within this community. Furthermore, the measurable metabolome was highly plastic to environmental variation.

## 1. Introduction

One of the greatest challenges to biologists is to understand the adaptive mechanisms of how organisms, at the individual, population and community level, will respond to climate change [1,2,3,4]. To achieve this, we need to understand the link between genetic variation and gross phenotypes by studying internal mechanisms that contribute to the development and survival of an individual within a community [5,6,7]. For example, changes in metabolism precede alterations in an organism’s morphology and, as such, metabolic traits can be considered a vital phenotype in wild populations and studied as the functional basis for adaptation [8,9]. Variation (plasticity) in the composition and concentration of metabolites may determine productivity, reproduction and ultimately survival and distribution of plant populations, especially those subjected to rapid climate change [10,11]. The application of metabolomics to understand the interactions between living organisms and the environment has formed the field of ‘environmental metabolomics’ [12,13,14,15]. Driven by advances in biochemical methodology, analysis of a global metabolism using metabolic fingerprinting approaches (rather than focussing on one metabolic trait) allows the determination of a general metabolic phenotype of a sample and identification of unexpected metabolite responses and pathway interactions that can be employed for discriminating samples based on their origin or their ecological relevance [16,17,18,19].

**Figure 1:**
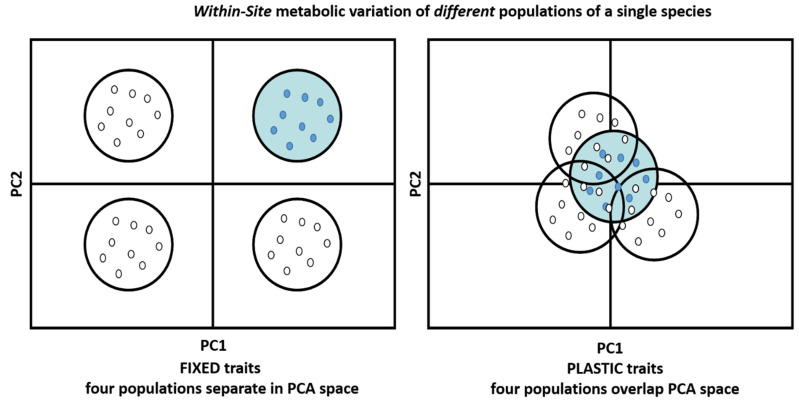
Hypothetical principal component analysis score scatter plots showing clustering of individual plant samples based on the metabolic phenotype profiles obtained from plants in a reciprocal transplant or common garden study. In this case, plants from four separate geographic populations were all grown within the same native location of one of the populations (this measures *within-site* variation). The native population location is termed “home” and the “away” populations are those plants introduced from another geographic population to the home location. In reciprocal transplant and common-garden studies, the within-site metabolic variation between the four different populations grown can be considered *fixed* if the metabolic traits of the away population (white, open) remain separate and dissimilar to the home population (blue) and *plastic* if clustered together and similar to the home population.

Environmental metabolomic approaches have previously been used to detect phenotypic variability at the metabolic level in response to environmental changes in drought [20,21], temperature [22,23,24,25], salinity [17,26], nutrient availability [27,28], and pollutants [29]. Inter and intraspecific comparisons of the metabolic composition of plants have also been made, finding for example variation in volatile leaf oils between *Eucalyptus* species [30], and intraspecific variation between genotypes of *Carex caryphyllea* [31] and the arctic-alpine species *Arabidopsis lyrata* spp. *petraea* [23,24,32]. Intraspecific variation in chemical defence is known to increase resistance towards hosts and herbivores [33,34,35]. The metabolic status of individuals within a community can indeed drive diversity and determine which species are able to survive within a community or niche, this being termed the “metabolic niche” [36,37,38]. Although single species metabolic studies are increasing in number, studies employing metabolomic techniques to address questions about the organisation of plant communities remain rare, but insightful. For example, Zuppinger-Dingley et al., [8] analysed the metabolic responses of individual plant species (grasses—*Festuca pratensis*, *Poa pratensis*; legumes—*Onobrychis viciifolia*, *Trifolium repens*; tall herbs—*Crepis biennis*, *Galium mollugo*; small herbs—*Plantago lanceolata*, *Prunella vulgaris*) to increasing plant diversity and found metabolic signatures differing between small and tall-statured species, and between mono-cultures and mixed species within an experimental plot, respectively. Assessing the variability in the metabolic phenotypic of multiple populations grown under common controlled-environmental conditions indicate whether the study species exhibit intra and interspecific metabolic variation. Field studies in environmental metabolomics are challenging. This is particularly the case when we move beyond the measurement of experimentally manipulated factors within sites to consider how metabolism varies at larger geographic scales. One field approach is through reciprocal transplant studies which test whether the local population (‘home’ populations) have greater fitness than populations transplanted to the local site (‘away’ populations). Fitness traits of home populations are usually expected to be optimised to local conditions [39]. The measurement of variation in traits, such as the metabolic phenotype, therefore provides information on whether local adaptation has occurred (Figure 1).

The objective of this study was to measure the interspecific and intraspecific metabolic variation, acclimation, and plasticity of plant species within a single community over a climatic gradient. This variation can be related to phenotypic plasticity (changes in phenotypes of a given genotype) or to genetic variability (or the interaction between both). Here, genetic variability is addressed by comparing the metabolic phenotypes of species and populations within species (from six different sites) grown under common conditions and phenotypic plasticity of the metabolic traits was investigated through reciprocal transplanting (see Figure 1 for a conceptual overview). While it could be expected that plants growing together in a community would share similar metabolic traits because they respond to the same environmental stimuli, this work tested the hypothesis that species within a local community would have different metabolic phenotypes to survive within a shared niche. The study system was based on communities of winter germinating annual plants located in grey dunes. Grey dunes are a well-defined fixed coastal habitat that exist in fragmented pockets along a latitudinal gradient of 1300 km from Portugal to England and exist in substantial variations in climate [40,41]. Five species of winter germinating annual plants were selected based on their membership of the same guild and their distribution across the study sites on our latitudinal gradient. We employ a metabolic fingerprinting approach using direct injection mass spectrometry (DIMS) to provide a snapshot overview of metabolic processes occurring within our plants at a particular point in time. We focus on identifying similarities and differences in metabolite masses to broadly assess chemical diversity in our populations and putatively identify these to suggest functions of these metabolites. The aim was to study multiple species in this dune community and populations in the common garden and reciprocal transplant study; however, due to adverse local climatic reasons only one species survived the latter field study.

## 2. Materials and Methods

### 2.1. Common-Garden Study (Inter and Intraspecific Metabolic Variation)

In our study, seeds of five dune annual species, *Arenaria serpyllifolia* (L.), *Cerastium diffusum* (L.), *Phleum arenarium* (L.), *Senecio vulgaris* (L.), *and Veronica arvensis* (L.) were collected from study sites along the Atlantic coast of Europe. The study sites were spread along a 1200 km latitudinal gradient, with differences in temperatures of 4 °C (average annual maximum) and 4.5 °C (average annual minimum) between the two most distant sites, and varied in a range of other environmental factors (Supplementary Table S1). Seeds were collected in the spring of 2009 from a pool of at least 20 mother plants in the field and were grown in the following conditions in 2012: twenty-five seeds (randomly selected) per species per population were sown in three pots containing Levington M3 compost (N204 P104 K339 ppm)/sand/vermiculite mixture (2:1:1) (6 × 6 × 10 cm pots, 25 seeds per pot). Pots were then placed in trays in controlled-environment growth rooms (Steil, 15 °C/10 °C day/night temperature cycle and 8/16 h light cycle). No additional nutrients were added to the substrate or water. At five weeks, seedlings were thinned to 12 individuals per pot. Leaf tissues were sampled (over a two-hour period during the middle of the photoperiod) at the same life history stage (rather than set number of days post germination) one week after the initiation of flowering in an individual plant. Five plants were sampled and pooled from each replicate pot producing 54 analytical samples (18× *Arenaria serpyllifolia* (6 populations), 9× *Cerastium diffusum* (3 populations), 15× *Phleum arenarium* (5 populations), 6× *Senecio vulgaris* (2 populations) and 6× *Veronica arvensis* (2 populations)) for mass spectrometry analysis. The variation in sample replicate number and population is due to not every species being present in every field population location (Table 1).

### 2.2. Metabolic Plasticity in the Field Using Reciprocal Transplants

Three dune annual species of high abundance and wide distribution along the latitudinal study gradient were sampled based on the ecological sampling methodology in [32,40,41,42]. *Arenaria serpyllifolia*, *Cerastium diffusum*, and *Veronica arvensis* were selected for analysis in the reciprocal transplant experiment. The same batches of seed collected in 2009 were used in this experiment. Twenty-five seeds per species per population were sown in three pots containing Levington M3 compost (N204 P104 K339 ppm)/sand/vermiculite mixture (2:1:1) (12 × 12 × 15 cm pots, 25 seeds per pot) and placed in trays in a controlled-environment growth cabinet as above and grown to seed. Seeds obtained from approximately 70 mother plants of each population were collected and bulked. In December 2010, seeds of each of the populations from plants grown under controlled environmental conditions were sown in mixed communities. Each community consisted of 15 *Arenaria* seeds, 15 *Veronica* seeds, and 20 *Cerastium* seeds, planted in 7 cm diameter circular pots using washed silica sand as a substrate. Seeds were germinated under controlled environmental conditions in a growth chamber as above. Seeds were germinated at varying success rates between different species and populations, and in February 2011, remaining communities of juvenile plants were transferred to five field sites (GB1, GB2, Fr1, Fr3, Fr4) (Supplementary Table S1). Each field site contained five replicate blocks located within 30 m of each other on flat terrain. Each replicate block contained five community pots, one from each source population. To avoid genetic contamination of local populations, plants had to remain in the pots and pots were removed from the sites before plants set seed. This resulted in the plants being exposed to more local microclimate than edaphic conditions for each site. Harvesting was conducted in April–May 2011 over a two-hour period at approximately midday. Tissue samples consisted of one leaf per individual plant taken from the third node. A summary of the surviving population and replicate numbers is given in Table 1.

### 2.3. Metabolite Quenching and Extraction

We used the boiling methanol quenching method for both the field and laboratory experiments. The excised leaf from each plant was immediately placed in a 1.5 mL screw cap plastic micro centrifuge tube, following which 0.5 mL of boiling 4:1 methanol–water (75 °C) was added to the tube. Tubes were placed in a warm water bath for three minutes to ensure that the methanol reached boiling point [13]. Although this is a valid method for metabolic quenching, due to new safety regulations, this method is now not recommended for field experiments [43,44]. The feasibility of liquid nitrogen quenching was explored, but the logistics of obtaining liquid nitrogen close to our field sites could not be resolved.

In the controlled environmental studies, tubes were then placed in a micro centrifuge box and stored in a freezer (−80 °C). In the reciprocal transplant field study, tubes were placed in a micro centrifuge box and kept cool in a freezer (−20 °C). Samples were sealed and secured before shipment by courier to the laboratory at the University of Sheffield where the samples were placed in a freezer (−80 °C). Time from sampling to storage at −80 °C in Sheffield ranged from 12 h to 4 days. In order to control for any degradation between sampling and analysis, a representative set of plant samples were spiked with known amounts of sucrose (20 μL of 40 mM sucrose).

Metabolites were extracted using a bi-phasic solvent system adapted from Davey et al. [23]. First, the 0.5 mL of methanol used in the sampling protocol was removed and placed into a 2 mL plastic tube. Leaf tissue samples were left intact and 300 μL of cold extraction solvent (methanol–water–chloroform, 2.5:1:1) was added to the sample. Tubes were vortexed and left on ice for 30 min with occasional shaking. This supernatant was then removed and added to the previous supernatant for the respective sample. This was repeated once more and then again using 600 μL chloroform as the extraction solvent. A total of 130 μL of cold distilled water was then added to each supernatant tube, which were then centrifuged (MSE Sanyo Hawk 15/05 refrigerated centrifuge) for 3 min (4 °C; 16,000× *g*) to separate the polar and nonpolar phases. Phases were removed into separate pre-labelled and chilled 2 mL tubes and stored at −80 °C. Sample tubes containing the remaining leaf tissue were placed in an oven at 50 °C for 3 h until the tissue was dry. The tissue was then weighed to determine dry weight. These dry weight data were then used to calculate dilutions for each sample in order to standardise the concentration of extracts across all samples.

### 2.4. Metabolite Fingerprinting—Direct Injection Mass Spectrometry (DIMS)

We used direct injection mass spectrometry (DIMS), a semi-quantitative non-separation chromatography technique which allows the high-throughput analysis of complex mixtures of metabolites, providing a sensitive tool used widely for broad scale metabolic phenotyping studies [23,32,45]. In both experiments, the aqueous methanol phase was directly infused into a QStar Pulsar I Mass Spectrometer (Applied Biosystems Sciex Instruments, Framingham, MA, USA) via a HP1090 Hewlett Packard HPLC autosampler. The HP1090 injected 25 μL of sample at a rate of 0.02 mL/min. Scan parameters were set to run for the duration of the injection: positive ionisation ToF MS scan with multi channel analysis (MCA) settings, mass range 50–1000 *m*/*z*. Samples were run in triplicate to allow for instrument error.

### 2.5. Metabolomic Analysis

DIMS studies produce spectra that detail the intensity of thousands of ‘features’, defined as atomic masses (recorded as mass to charge (*m*/*z*) ratios) that represent different metabolites [46]. Raw centroid total ion count (TIC) peak mass data from the mass spectrometer were aligned and binned at 0.2 Da *m*/*z* units (and converted to %TIC) using an in-house excel macro as described in [47] and modified by MM Burrell (author). Percent total ion count (% TIC) data for each bin of each sample were analysed by principal component analysis (PCA) using Simca-P (Version 12, Umetrics, Sweden) software. Data were normalised by Pareto-scaling within Simca-P to reduce the potentially biasing influence of larger spectral masses. Analysis of variance (ANOVA) was applied to each detected binned mass in order to determine the statistical significance of the metabolic differences between samples. Factors tested were species and population of origin. The application of univariate analyses to metabolomic data may result in high numbers of false positive results [48], especially given the low (*n* = 3) sample replicate number. Therefore, false detection rate (FDR) corrections were used to correct *p*-values from ANOVAs. The list of significantly different bins according to species was combined with the list of masses positively contributing to species separation in PCA.

### 2.6. Putative Identification and Metabolite Mapping

Putative identifications were based on the mean mass of the accurate masses detected within a mass “bin” of interest (0.2 Da bins). When variance, measured as standard deviation of the mass within the bin, was greater than 0.05, the list of accurate masses was consulted to determine whether the bin contained two ion peaks. In cases such as this, the accurate masses were split into groups and the average of each peak calculated. Putative identifications based on these averages of accurate masses detected were assigned compound names from KEGG compound (http://www.genome.jp/kegg/, accessed on 13 January 2025), an online database (MZedDB) [49], using an excel macro developed by MM Burrell—co-author). These putative identifications were analysed using the MetaboAnalyst v2.0 (www.metaboanalyst.ca) [50] functional interpretation tool, to detect potential key pathways that were up-regulated or down-regulated in different sample classes. The putatively annotated metabolites were not individually or further quantified. While these identifications are speculative, putative identification and pathway analysis are an important step in phenotyping analyses and will allow us to design further experiments to confirm identifications using tandem mass spectrometry (MS/MS) and quantify differences between samples.

## 3. Results

### 3.1. Inter-Specific Variation in Metabolic Phenotypes of Sand Dune Species in Common Garden

**Figure 2:**
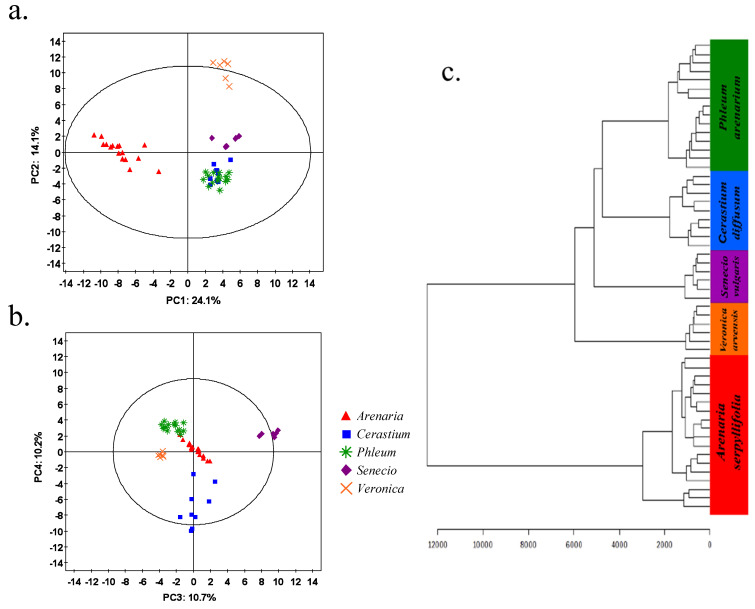
Inter-specific metabolic variation. Score scatter plots (Figure 2a = PC1 and PC2; Figure 2b = PC3 and PC4) from principal component analysis of *m*/*z* values (binned to 0.2 Da) obtained by DIMS (positive ionisation) metabolic fingerprinting of the methanol/aqueous phase from five sand dune plant species, *Arenaria serpyllifolia* (red triangles), *Cerastium diffusum* (blue squares), *Phleum arenarium* (green stars), *Senecio vulgaris* (purple diamonds), and *Veronica arvensis* (orange crosses). The percent of the variation in the data explained by each component is provided in each graph. Figure 2c shows the above dataset presented as a hierarchical cluster analysis built on the eigenvalues coordinates from combined PC1 to PC4.

We found strong evidence of interspecific separation of species in metabolic space. Masses detected within the aqueous phase analysed in positive ionisation mode showed clear clustering of each species (Figure 2a,b). PCA scores plots indicate that *V. arvensis* and *A. serpyllifolia* samples both clustered away from other species along PC2 and PC1, respectively (Figure 2a). *Senecio vulgaris* and *C. diffusum* clustered along PC3 and PC4, respectively, while *P. arenarium* clustered between these two components (Figure 2b). Using the same binned dataset, a dendrogram can be built (Euclidean distance) on the combined eigenvalues of the samples in PC1 to PC4 to measure the similarity and relatedness of each species (Figure 2c). In common with Figure 2a, *P. arenarium* and *C. diffusum* were within the same node (Figure 2a) with *A. serpyllifolia* behaving as an outgroup to all the other species.

**Figure 3:**
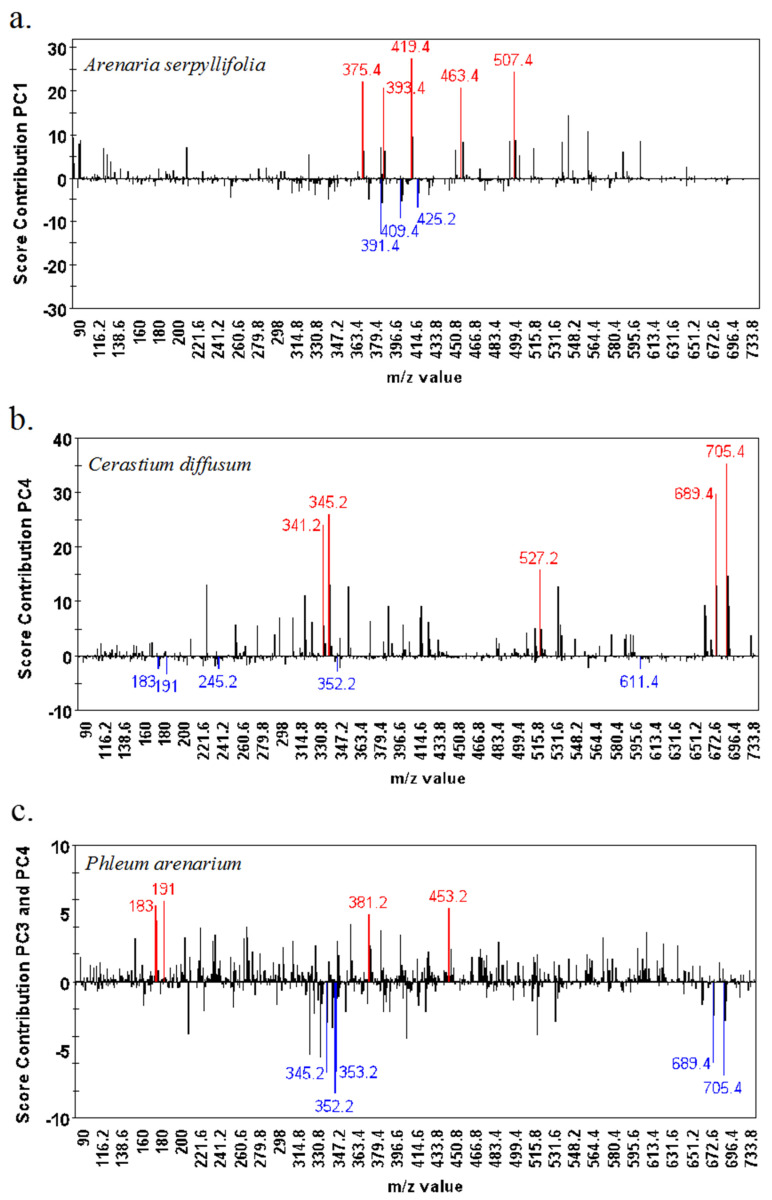

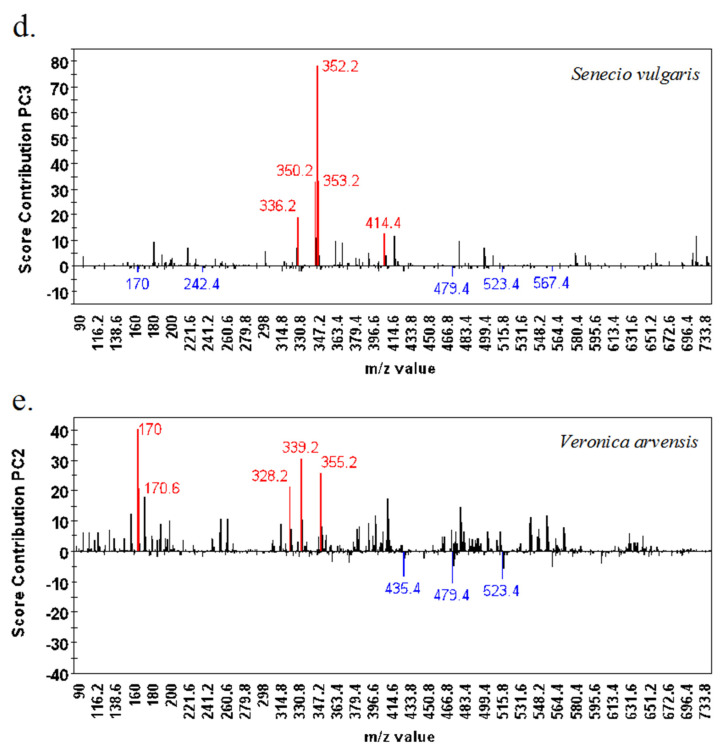
Score contribution plots relating to principal component analysis of *m*/*z* values (binned to 0.2 Da) obtained by DIMS of the aqueous/methanol phase. Plots indicate which bins are in high abundance (RED, more positive) and low abundance (BLUE, more negative) in (**a**) *Arenaria serpyllifolia*, (**b**) *Cerastium diffusum*, (**c**) *Phleum arenarium*, (**d**) *Senecio vulgaris*, and (**e**) *Veronica arvensis* along the principal components shown to correspond to species separation.

The scores contribution plots (that correspond directly to the PCA plots shown in Figure 2) indicate which mass bins are in high abundance (more positive) and low abundance (more negative) for each species in relation to all other species analysed (Figure 3a–e). Each species has a unique metabolic phenotype in that the composition of the main discriminating mass bins are different for each species. For example, *C. diffusum* and *A. serpyllifolia* samples have high abundances of masses in the high range (>300 *m*/*z*) (Figure 3a,b); *P. arenarium* and *V. arvensis* are typified by large quantities of low-mid range masses (between 150 and 200 *m*/*z*) (Figure 3c,e), while the mass bin 352.2 is found to highly discriminate *S. vulgaris* (Figure 3d).

**Figure 4:**
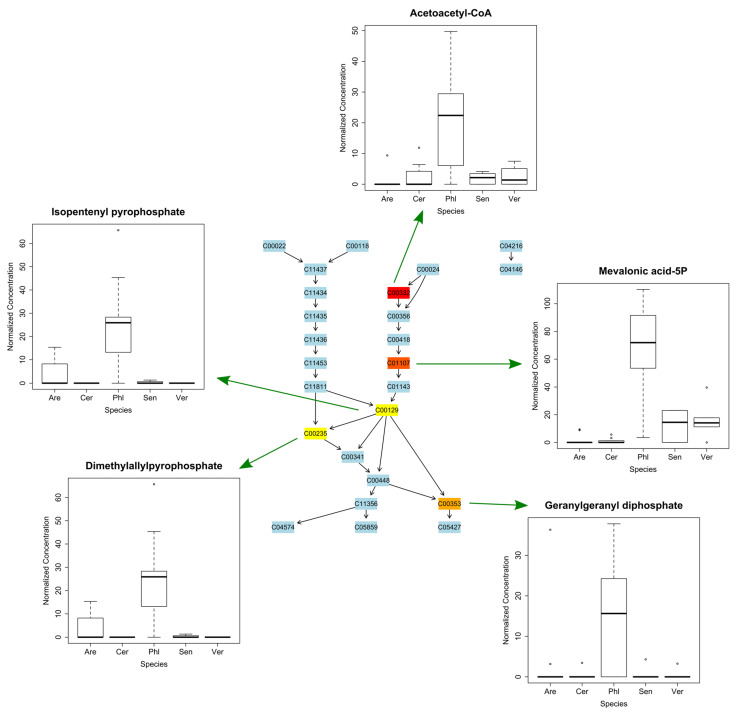
An example of metabolic mapping pathway analysis using Metaboanalyst (http://www.metaboanalyst.ca/). Pathway of terpenoid backbone metabolism, putatively identified metabolites highlighted from yellow to red were found to be significantly higher in abundance in *Phleum arenarium* (Phl) compared to the other sand dune species. Dots indicate outliers in the dataset.

There were 523 bins (0.2 Da range bins) that had significantly different average ion counts between all species, with a *p*-value below 0.05 (FDR corrected *p*-value). The resulting Tukey’s pot-hoc test showed that *Veronica arvensis* had the highest number of uniquely higher bins (222 bins); 70, 51, 38, and 43 bins were uniquely higher in *P. arenarium*, *S. vulgaris*, *C. diffusum*, and *A. serpyllifolia*, respectively. The top two putatively identified metabolites that discriminated *A. serpyllifolia* were ergosterol and lupeol; for *C. diffusum*, they were kaurenoic acid and clusianose; for *P. arenarium*, they were sorbitol and homogentisate; for *S. vulgaris*, they were senecionine and riddelliine; and for *V. arvensis*, they were 5-amino-levulinate and sphinganine (Supplementary Table S2). Metabolite pathways of the putatively identified metabolites associated with each population were mapped onto pathways using MetaboAnalyst mapping software (Supplementary Table S3). Overall, the top pathways identified as positively discriminating each species were related to amino acid metabolism in *A. serpyllifolia* and *V. arvensis*; carbohydrate metabolism discriminating *C. diffusum*; lipid metabolism for *S. vulgaris*; and a mix of pathways for *P. arenarium*. *P. arenarium* was the only species in which discriminatory metabolites mapped onto pathways were involved in terpene and polyketoid metabolism (Figure 4). Specific metabolic pathways that differed the most between species were the arginine and proline pathway in *A. serpyllifolia*; amino sugar and nucleotide sugar metabolism in *C. diffusum* and *P. arenarium*; biosynthesis of unsaturated fatty acids in *S. vulgaris*; and purine metabolism in *V. arvensis*.

### 3.2. Intra-Specific Variation in Metabolic Profiles of Sand Dune Species in Common Garden

**Figure 5:**
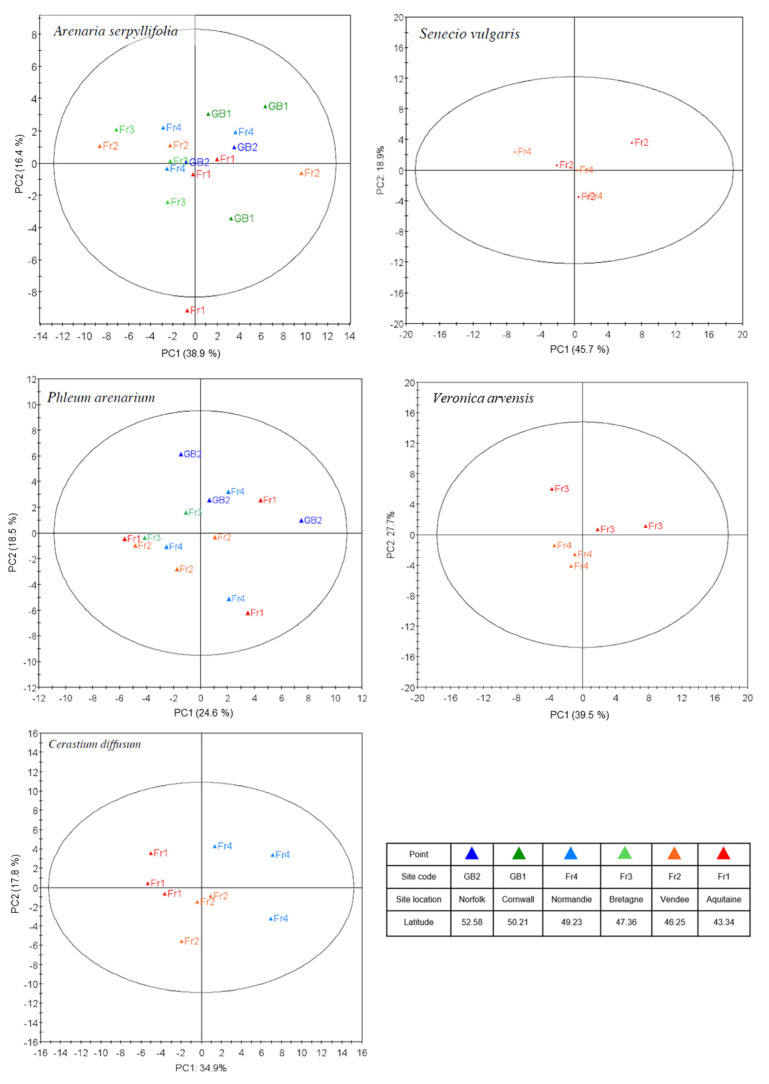
Intra-specific metabolic phenotype variation. Score scatter plots from principal component analysis of *m*/*z* values (binned to 0.2 Da) obtained by metabolic fingerprinting populations of five species grown under identical conditions in controlled environment cabinets, *A. serpyllifolia*, *P. arenarium*, *S. vulgaris*, *V. arvensis*, and *C. diffusum*. Legend provides location information for each population of origin. Percentage of the variation in the data explained by each component is provided. *n* = 3 per population.

In contrast with the interspecific patterns, we found no intraspecific clustering of the metabolic phenotype of three out of the five species, *A. serpyllifolia*, *P. arenarium*, and *S. vulgaris* (Figure 5), indicating that the metabolic phenotype expressed by each population is relatively similar. However, *V. arvensis* populations Fr3 and Fr4 showed some separation along PC2 and *C. diffusum* showed strong clustering along PC1 according to population. Univariate statistical analyses (ANOVA) were then applied to determine whether the detected abundance of each mass bin statistically differed according to population before putative identifications of these mass bins were made (Supplementary Table S4). After the false detection rate (FDR) corrected *p*-value was determined, a number of mass bins that had abundances varying significantly according to population were identified. In *A. serpyllifolia*, 8 bins differed between populations (putatively identified as guanosine, 4-aminobutyraldehyde, scopolamine, aspartate, GA24, muramic acid, elaidic acid), 13 bins differed between populations of *C. diffusum* (putatively identified as lauric acid, falcarinidiol, sphinganine, kaurenic acid, retinal, guanidinobutyrate, xanthosine, isopentyladenine), 1 bin differed between populations of *P. arenarium* (putatively identified as oleic acid), but no bins differed between populations of *S. vulgaris* and *V. arvensis*.

### 3.3. Metabolic Plasticity in Field Reciprocal Transplants

**Figure 6:**
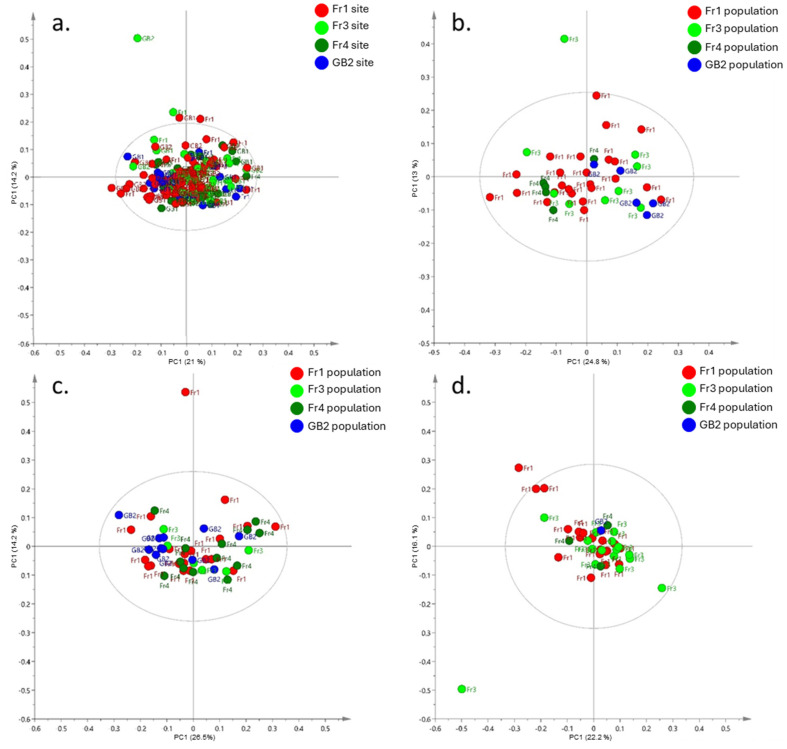
Score scatter plots from principal component analysis of *m*/*z* values (binned to 0.2 Da) obtained by metabolic fingerprinting *V. arvensis* from the reciprocal transplant study between GB1, GB2, and Fr2 sites. (**a**) Between-site variation PCA of all reciprocal transplant data (coloured according to plant site origin and labelled according to plant growth location); (**b**) within-site variation in *V. arvensis* from Fr1, Fr3, Fr4, GB2 growing in site Fr1; (**c**) within-site variation in *V. arvensis* from Fr1, Fr3, Fr4, GB2 growing in site GB1; (**d**) within-site variation in *V. arvensis* from Fr1, Fr3, Fr4, GB2 growing in site GB2. Figure 6b–d are coloured and labelled according to plant site origin.

The time period in which the reciprocal transplant was conducted was exceptionally dry, therefore reducing the number of all populations surviving in all transplant sites. A summary of the surviving population and replicate numbers is given in Table 1. This resulted in *V. arvensis* being the only species to survive, with the majority of the samples from the Fr1 site surviving in all three transplant locations (Fr1, GB1, and GB2). From the samples that were available for harvest and subsequent metabolic analysis, there were no discernible clustering of samples based on the origin of the plants or where they were growing (Figure 6a–d). For example, Fr3, Fr4, and GB2 plants growing at the Fr1 site had metabolic profiles similar to the Fr1 plants resulting in an overlap of samples in PCA space (Figure 6b), indicating that the metabolic phenotype was influenced more by its local growth environment than its original genotype.

## 4. Discussion

### 4.1. Interspecific Variation in Metabolic Phenotypes

The metabolic profiles of different species growing under the same ambient environment suggest that although they experience similar biotic and abiotic conditions, contrasting metabolic functions are produced at a similar life history stage. The metabolic mapping of the putatively identified metabolites indicated that although the plant species were up-regulating different compounds (Supplementary Table S2), there were ontological similarities in the pathways that those compounds were related to (Supplementary Table S2) with many of the compounds mapped onto pathways relating to primary metabolism. For example, the majority of pathways showing compounds up-regulated in *C. diffusum*, are involved in carbohydrate metabolism, and the identified pathways for *V. arvensis* are associated with amino acid metabolism. Primary metabolism, involving the initial assimilation of carbon via photosynthesis and nitrogen, will show basic similarities across all species because these are essential functions enabling plants to survive. A number of compounds causing positive discrimination of *V. arvensis* were putatively identified as Gibberellins (Supplementary Table S2). Gibberellins (GAs) are a group of terpenes that typically act as growth promotors and regulators [51,52]. Abiotic factors such as low temperature, salt stress, and high temperature are known to cause differential expression of gibberellin oxidase genes that encode gibberellin compounds [53], and can in turn affect the initiation of flowering in plants [54].

In *V. arvensis*, leaf samples for metabolomics analysis were collected one week after initial flowering and, as growth analysis indicates that flowering coincides with a rapid increase in height in *V. arvensis*, it may explain why the growth promoting compounds, GAs, positively discriminate this species. Many of the important *m*/*z* value bins statistically separating *Senecio vulgaris* from the other species had putative identifications that fell into the class of compounds known at the pyrrolizidine alkaloids (PAs). These secondary compounds are particularly numerous and diverse in the *Senecio* genus [55] and have been widely studied due to their hepatoxic effect on livestock and humans [56]. The putative identification of pyrrolizidine alkaloids up-regulated in *S. vulgaris* corroborates other evidence that there can be strong phylogenetic constraints on metabolic variation. It has, however, been more difficult to attribute metabolic variation in the other species to patterns of relatedness according to their phylogeny. Analysing whether species show metabolic divergence in relation to their geographic origin provides the next step in understanding the importance of genetic adaptation.

### 4.2. Intraspecific Variation in Metabolic Phenotypes

Visualisation of metabolic similarities and differences between plant samples indicated that plant metabolic phenotype varies depending on the population as well as the species studied. Plants respond to environmental stresses via rapid and reversible alterations in their metabolic compounds, which is the primary mechanism by which plants acclimate to rapidly changing environmental conditions and forms the basis of physiological plant homeostasis [57]. The flexibility in the metabolic expression in *A. serpyllifolia*, *P. arenarium*, and *S. vulgaris* implies that populations of these species maintain metabolic plasticity rather than metabolic adaptation to home conditions. Significant metabolic differences were picked up in the Fr1 population of *A. serpyllifolia* and many of the putative identifications of these compounds mapped onto pathways relating to amino acid metabolism. There are examples of metabolomic phenotype studies that have identified accumulations of various amino acids in response to environmental stresses including drought, salt, temperature, and heavy metals [58,59,60]. Amino acids are known to accumulate in response to different environmental stresses. The differentiation of *A. serpyllifolia* population Fr1 in relation to amino acid biosynthesis is therefore likely to be occurring due to constitutive high expression of these compounds, rather than a response to environmental stress. A review of responses to heavy metal stress [61] highlights three species (including the perennial herb *Silene vulgaris* belonging to the Caryophyllaceae) known to have metal-tolerant populations that express substantially elevated constitutive proline levels, even in the absence of excess metal ions when compared to non-tolerant populations. Our Caryophyllaceae species *A. serpyllifolia* may be exhibiting tolerance to drought in the Fr1 population, that is not constitutively expressed in other populations.

### 4.3. Reciprocal Transplants—Local Adaptation and Metabolic Plasticity

A key task for metabolomic research is to understand how much of the variation in the metabolome is influenced by inherent natural genetic versus external environmental factors [13,62]. Our objective for carrying out the reciprocal transplant study was to assess a population’s adaptation to its local environment and provide a system to test whether the measurable metabolic traits are largely fixed or plastic. Unfortunately, high mortality of the reciprocally sown plants resulted in data only being available for one species, *Veronica arvensis*, so care needs to be taken when making generalisations. The fact that only one species did survive does still provide valid information on the variance in the resiliance for transplanting and survival of such species in these ecosystems. Nevertheless, the results showed that there were no discernible clustering of samples based on the origin of the plants or where they were growing (Figure 5a–d). Using the hypothetical model in Figure 1, it is apparent that the metabolic phenotypes obtained from all samples were similar across all sites regardless of where the plants originated from. This implies that the metabolome is highly plastic and that the measurable metabolome in this study is influenced more by local environmental factors than inherent genetic factors that are within the genetic variation pool of the samples selected. This is in line with other studies on plant systems such as the Arctic-Alpine species *Arabidopsis lyrata* ssp. *petraea* that also showed increased metabolic plasticity in outdoor common garden experiments [63] but does present distinct metabolic separation between locations based on single and multiple temperature treatments in controlled environment growth cabinets [23,24]. In order to make more concrete conclusions (given that only one species survived and it was an exceptionally dry year), such field experiments will have to be repeated using greater numbers of individual plants from more species in order to ensure the survival of more plants for the metabolite harvesting. An alternative approach would be to use controlled simulations in a common garden experiment [23,24]. While only a limited number of environmental factors could be adjusted (temperature and rainfall), it could help ameliorate the implications of extreme adverse conditions on a metabolomic study such as this.

## 5. Conclusions

Metabolically phenotyping five species of winter annual plants showed that species living together in a community have distinct metabolic phenotypes, while analysis of metabolic trait variation between populations growing under standard environmental conditions elicited species-specific responses. The metabolic variation measured in the plants under the reciprocal transplant study was high. The results showed that the metabolic phenotypes obtained from all samples were similar across all sites regardless of where the plants originated from. This implies that the metabolome is highly plastic and that the measurable metabolome in this study is influenced more by local environmental factors than inherent genetic factors. The underlying genetic contribution to this variation would need to be further investigated. Our work has highlighted that species are fulfilling different niches within these sand dune community. There are practical implications of the findings for conservation or restoration of sand-dune systems. We have identified that there is species-specific resilience when transplanting these species, and establishment even under semi-controlled environments is challenging. However, those that do survive appear to have similar and plastic metabolic phenotypes and although speculative until further data are obtained, that could play a role in the increased survival rate of those that are transported for restoration purposes.

## Figures and Tables

**Table 1:** Population and replicate number (in parenthesis) for the common garden-controlled environment and reciprocal transplant studies. GB = Great Britain. Fr = France.

Species	Common Garden *	Reciprocal Transplant
*Arenaria serpyllifolia*	GB1, GB2, Fr1, Fr2, Fr3, Fr4 (3)	None survived
*Cerastium diffusum*	Fr1, Fr2, Fr4 (3)	None survived
*Phleum arenarium*	GB2, Fr1, Fr2, Fr3, Fr4 (3)	Not selected for experiment
*Senecio vulgaris*	Fr2, Fr4 (3)	Not selected for experiment
*Veronica arvensis*	Fr3, Fr4 (3)	In site Fr1 = Fr1 (24), Fr3 (9), Fr4 (5), GB2 (5) In site GB1 = Fr1 (20), Fr3 (6), Fr4 (13), GB2 (11) In site GB2 = Fr1 (21), Fr3 (17), Fr4 (3), GB2 (1)

* each replicate consists of five pooled plants per pot.

## Data Availability

Metabolite data (0.2 da bins, %TIC of samples) are available to download under Supplementary Materials/supporting information (Supplementary File S1). In addition the data are also provided in the PhD Thesis by R.G. at https://etheses.whiterose.ac.uk/10777/ (accessed on 13 January 2025).

## Supplementary Materials

The following supporting information can be downloaded at: https://www.mdpi.com/article/10.3390/metabo15040217/s1. Supplementary Table S1. Location of field sites studied with a summary of climate, topography, and grazing intensity. Supplementary Table S2. Summary of the top putative identifications for mass bin values that positively and negatively contribute to species separation as shown in the PCA score scatter plot (Figure 2). Supplementary Table S3. Metabolite pathways of the putatively identified metabolites associated with each species. Supplementary Table S4. Table showing which mass bins contain compound peaks that significantly differ according to population of origin. Supplementary File S1. Excel file of 0.2 da *m*/*z* bins and associated %total ion count (TIC) for all samples.

## Abbreviations

Da (Dalton); DIMS (Direct Injection Mass Spectrometry); Fr (France); GB (Great Britain); PCA (Principal Component Analysis); %TIC (Percent Total Ion Count).
