# Supplementary material for: Metabolic Niches and Plasticity of Sand-Dune Plant Communities Along a Trans-European Gradient
Source: Metabolites. 2025 Mar 24;15(4):217. doi: 10.3390/metabo15040217 (PMC12029026; doi:10.3390/metabo15040217)
Supplement: Supplementary file 1 [file metabolites-15-00217-s001.zip › Supplementary Tables.pdf]

Supplementary Table S1. Location of field sites studied with a summary of climate, topography and grazing intensity. Mean annual rainfall and temperature values are given for the study period 2008-2011. Grazing intensity represents counts of rabbit fecal pellets per m^2. Mean values (± SD) for slope, moss & lichen cover and depth, and grazing intensities are based on m^2 quadrat surveys (*n=145-302*).

Site location (site code)	Latitude	Longitude	Average annual Max/Min Temp. (°C)	Annual Rainfall (mm)	Degree of Slope	Moss and Lichen		Grazing Intensity
						% cover	Depth (mm)	
**Holkham,** Norfolk, UK (GB2)	52°58'604"N	0°46'033"E	13.8/5.7	621.3	6.3 ± 4.9	87±18	1.8 ± 1.0	High (85)
**Penhale,** Cornwall, UK (GB1)	50°21'716"N	5°08'316"W	13.3/7.9	1042.6	14.5 ± 6.0	85±18	2.2 ± 0.6	Very High (165)
**Hatainville,** Normandie, France (Fr4)	49°23'854"N	1°49'231"W	14.7/7.1	723.2	7.4 ± 5.0	80±22	1.3 ± 0.6	High (72)
**Sainte Barbe,** Bretagne, France (Fr3)	47°36'021"N	3°08'858"W	15.5/7.9	927.4	3.3 ± 3.4	88±21	3.3 ± 1.1	High (86)
**Les Conches,** Vendee, France (Fr2)	46°25'523"N	1°29'721"W	16.6/9.5	763.5	6.9 ± 5.3	67±32	1.9 ± 1.3	Medium (31)
**Ondres Plage,** Aquitaine, France (Fr1)	43°34'610"N	1°29'055"W	17.8/10.2	1510	0.1 ± 0.9	69±31	1.6 ± 0.8	Medium (35)

Supplementary Table S2. Summary of the top putative identifications for mass bin values that positively and negatively contribute to species separation as shown in the PCA score scatter plot (Fig. 2). Identifications highlighted here are chosen based on similarity to the average mass value and known occurrence in the plant species.

	Mass Bin	PCA Score Contribution	Average Mass Value	Putative Identification
a) Arenaria serpyllifolia				
Positively contribute	419.4	26.652	419.3596	Ergosterol, 6-Alpha-Hydroxycampestanol, 6-Deoxocathasterone
	507.4	23.793	507.4044	Lupeol acetate
	375.4	21.541	375.3153	Prostaglandin-H2
	393.4	20.268	393.3736	5-Dehydroavenasterol
	463.4	20.23	463.3810	Nonacosan-10-ol
Negatively contribute	391.4	-12.582	391.3698	Prostaglandin-H2
	409.4	-9.27	409.3631	Cholesterol
	425.2	-6.424	425.2057	Robustadial A
	392.4	-5.507	392.3840	Dopaxanthin
	410.4	-5.218	410.3919	Veratramine, Benzyladenine-7-N-Glucoside, Benzyladenine-9-N-Glucoside
b) Cerastium diffusum				
Positively contribute	341.2	22.453	341.2818	Ent-Kaurenoic Acid, Ent-7-Alpha-Hydroxykaurenoic Acid, 3'-Ketolactose, Leukotriene A4
	345.2	22.969	345.1880	Clusianose, Maltitol, Melibiitol
	527.2	13.321	527.2688	Eudesobovatol A, Asiatic acid
	689.4	26.695	689.3486	Callichiline
	705.4	31.744	705.3300	Cularine, S-Tetrahydrocolumbamine, Ajmalicine
Negatively contribute	183	-1.769	183.0085	2-Oxoadipate, Tricholomic acid
	191	-2.752	191.0954	3-Dehydroquinate, Pyridoxamine, 3-Sulfinyl-Pyruvate, L-Alpha-Amino-y-Oxalyaminobutyric Acid
	245.2	-2.639	245.1846	Beta;-L-Fucose, Falcarinol, N1-Acetylspermine
	352.2	-6.022	352.2270	Retrorsine, Senecionine N-oxide, Reticuline
	611.4	-2.044	611.4263	2-Ketospirilloxanthin

Supplementary Table S2. Continued

	Mass Bin	PCA Score Contribution	Average Mass Value	Putative Identification
c) Phleum arenarium				
Positively contribute	183	5.288	183.0855	Sorbitol, 4-Guanidino-Butanamide, Tryptamine, Mannitol
	191	5.044	191.0777	Homogentisate, Xylitol, Pyridoxamine, 3-dehydroquinate
	365.2	5.414	365.1835	Ajmaline, GA44, Xanthosine-5-Phosphate
	381.2	5.653	381.1620	Pinoresinol
	453.2	6.565	453.2183	Athamantin
Negatively contribute	170	-4.504	170.0916	5-Amino-Levulinate, Glutamate-1-Semialdehyde, L-Proline, Leucine
	350.2	-3.621	350.2050	Riddelline, Spiradine A
	352.2	-8.757	352.2077	Retrorsine, Senecionine N-oxide, Reticuline
	353.2	-8.226	353.2026	Geranyl-Diphosphate, 5-phosphoribosyl-N-formylglycineamide, Prostaglandin E2, 7,8-Dihydropteroate
	414.4	-5.363	414.4052	Dopaxanthin
d) Senecio vulgaris				
Positively contribute	336.2	17.003	336.1398	Senecionine, Spiroxamine, S-methyl-5'-thioadenosine
	350.2	29.301	350.2012	Riddelline, Spiradine A
	352.2	68.682	352.1347	Retrorsine, Senecionine N-oxide, Reticuline
	353.2	32.853	353.1570	Geranyl-Diphosphate, 5-phosphoribosyl-N-formylglycineamide, Prostaglandin E2, 7,8-Dihydropteroate
	414.4	13.6	414.3719	Dopaxanthin
Negatively contribute	170	-2.031	170.0518	5-Amino-Levulinate, Glutamate-1-Semialdehyde, L-Proline, Leucine
	242.4	-3.21	242.3733	-
	479.4	-5.104	479.3887	24-Methylcycloartenol
	523.4	-3.388	523.4133	-
	567.4	-3.001	567.4439	Phytoene
e) Veronica arvensis				
Positively contribute	170	41.009	170.0976	5-Amino-Levulinate, Glutamate-1-Semialdehyde, L-Proline, Leucine
	170.6	21.281	170.5987	Sphinganine, dTMP, Leucodelphinidin
	328.2	21.865	328.1799	Hyoscyamine
	339.2	32.233	339.1829	Gibberellin A9, GA12-Aldehyde, N5-Carboxyaminoimidazole, Aminoimidazole
	355.2	26.358	355.2798	Gibberellin A9, GA12-Aldehyde, Gibberellin A4, Gibberellin A12
Negatively contribute	435.4	-8.237	435.3717	Stigmasterol, 6-Deoxotyphasterol
	479.4	-10.445	479.3864	Flavin Mononucleotide
	523.4	-9.089	523.4175	-
	524.4	-5.501	524.4145	-
	567.4	-5.287	567.4126	Phytoene

Supplementary Table S3. Metabolite pathways of the putatively identified metabolites associated with each species. Pathways of interest based on the most discriminatory mass bins determined by ANOVA. Putative identifications of the accurate mass contained within each mass bin was first determined and then uploaded onto MetaboAnalyst mapping software. http://www.metaboanalyst.ca/ Pathways are ranked in descending order of importance based on the total number of metabolites within a pathway and the number of identified metabolites within the pathway that were identified.

Species	Pathway	Total Compounds in Pathway	Compounds in Pathway Significantly Different	Proportion of Pathway Significantly Different	Raw p-value	FDR	Impact
Arenaria serpyllifolia	Arginine and proline metabolism	38	5	0.13	2.92E-10	1.23E-08	0.317
	Cyanoamino acid metabolism	11	4	0.36	6.02E-08	1.57E-07	0
	Pyruvate metabolism	21	3	0.14	2.99E-09	1.40E-08	0.116
	Alanine, aspartate and glutamate metabolism	22	3	0.14	1.14E-09	1.40E-08	0.603
	Glycolysis or Gluconeogenesis	25	3	0.12	2.99E-09	1.40E-08	0.001
	Cysteine and methionine metabolism	34	3	0.09	6.2E-09	2.09E-08	0.022
Cerastium diffusum	Amino sugar and nucleotide sugar metabolism	41	3	0.07	2.15E-08	6.03E-08	0.121
	Pentose and glucuronate interconversions	12	2	0.17	8.85E-10	5.90E-09	0.000
	Ascorbate and aldarate metabolism	15	2	0.13	8.85E-10	5.90E-09	0.000
	Starch and sucrose metabolism	30	2	0.07	3.20E-11	6.39E-10	0.042
	Biosynthesis of unsaturated fatty acids	42	2	0.05	1.56E-08	5.20E-08	0.000
Phleum arenarium	Amino sugar and nucleotide sugar metabolism	41	3	0.07	3.69E-09	1.08E-07	0.095
	Pentose phosphate pathway	18	3	0.17	5.25E-08	2.06E-07	0.426
	Zeatin biosynthesis	19	3	0.16	5.56E-08	2.06E-07	0.396
	Terpenoid backbone biosynthesis	25	3	0.12	5.84E-09	1.08E-07	0.098
	Arginine and proline metabolism	38	3	0.08	1.50E-08	1.39E-07	0.130
Senecio vulgaris	Biosynthesis of unsaturated fatty acids	42	2	0.05	1.53E-07	3.07E-07	0.000
	Purine metabolism	61	2	0.03	3.32E-11	3.98E-10	0.000
Veronica arvensis	Purine metabolism	61	7	0.11	1.10E-12	1.33E-11	0.163
	Alanine, aspartate and glutamate metabolism	22	5	0.27	1.01E-13	4.93E-12	0.339
	Arginine and proline metabolism	38	5	0.16	1.63E-12	1.33E-11	0.163
	Histidine metabolism	16	4	0.25	1.52E-12	1.33E-11	0.488
	Aminoacyl-tRNA biosynthesis	67	4	0.06	6.07E-13	1.33E-11	0.000

Supplementary Table S4. Table showing which mass bins contain compound peaks that significantly differ according to population of origin. Statistical significance is determined by False Detection Rate (FDR) values below 0.05. Details of the direction of the compound abundance difference are provided.

Species	Mass Bin	Average Accurate Mass	p-value	FDR	Direction of concentraction difference		Putative Identifications
A. serpyllifolia	266.2	266.1382 / 266.2507	4.544E-06	0.0028	Fr1	↑	Guanosine, Abscisic Aldehyde, Xanthoxin, Dihydrozeatin
A. serpyllifolia	110	110.050	4.187E-05	0.0097	Fr1	↑	4-Aminobutyraldehyde
A. serpyllifolia	302.2	302.209	4.771E-05	0.0097	Fr1	↑	Scopolamine, N-Acetyl-Alpha;-D-Galactosamine, N-Acetyl-Glucosamine-1-Phosphate
A. serpyllifolia	134	134.061	8.650E-05	0.0132	Fr1 and GB2	↑	Aspartate, Cytosine, Histamine
A. serpyllifolia	369.2	369.186	1.600E-04	0.0195	Fr1 and Fr3	↑	GA24, Prostaglandin G
A. serpyllifolia	252.2	252.143	2.744E-04	0.0253	Fr1	↑	Muramic acid, Deoxyadenosine
A. serpyllifolia	321.2	321.206	2.901E-04	0.0253	Fr1 and GB2	↑	Elaidic acid, Oleic acid
A. serpyllifolia	126	126.066	5.304E-04	0.0405	Fr1 and GB2	↑	4-Aminobutyraldehyde, GABA
C. diffusum	201.2	201.157	1.970E-05	0.0074	Fr4	↑	Lauric acid, 4-Fumaryl-Acetoacetate,
C. diffusum	261.2	261.169	4.422E-04	0.0254	Fr4	↑	Falcarinidiol, D-Hexose,D-Mannose-6-Phosphate, Inositol, D-Fructose-6-Phosphate
C. diffusum	274.4	274.338	3.165E-04	0.0233	Fr4	↑	No ID Available
C. diffusum	284.4	284.324	1.027E-04	0.0130	Fr4	↓	Sphinganine
C. diffusum	305.2	305.210	9.269E-04	0.0451	Fr4	↑	Ent-kaurenic acid, Oleic acid
C. diffusum	307.2	307.186	1.007E-04	0.0130	Fr4	↑	Retinal, Stearic acid
C. diffusum	308.2	308.200	3.899E-04	0.0246	Fr4	↑	4-Guanidinobutyrate, Hexanedioic acid
C. diffusum	341.2	341.184	3.322E-04	0.0233	Fr4	↓	Ent-kaurenoic acid, Leukotriene A4
C. diffusum	387.2	387.237	4.819E-05	0.0102	Fr4	↑	Xanthosine-5-phosphate
C. diffusum	404.4	404.320	5.649E-04	0.0298	Fr4	↑	Isopentenyladenine-9-N-glucoside
C. diffusum	516.2	516.259	2.790E-04	0.0233	Fr1	↑	No ID Available
C. diffusum	597.8	597.803	2.343E-05	0.0074	Fr1	↑	No ID Available
C. diffusum	678.8	678.824	2.658E-04	0.0233	Fr1	↑	No ID Available
P. arenarium	305.4		2.106E-07	0.0002	Fr4	↑	Oleic acid
S. vulgaris	-		-	-	-	-	
V. arvense	-		-	-	-	-	
